# Moroccan Propolis: A Natural Antioxidant, Antibacterial, and Antibiofilm against* Staphylococcus aureus* with No Induction of Resistance after Continuous Exposure

**DOI:** 10.1155/2018/9759240

**Published:** 2018-11-12

**Authors:** Soukaïna El-Guendouz, Smail Aazza, Badiaa Lyoussi, Vassya Bankova, Milena Popova, Luís Neto, Maria Leonor Faleiro, Maria da Graça Miguel

**Affiliations:** ^1^Laboratory of Physiology-Pharmacology-Environmental Health, Faculty of Sciences Dhar El Mehraz, BP 1796 Atlas, University Sidi Mohamed Ben Abdallah, Fez 30 000, Morocco; ^2^Department of Chemistry and Pharmacy, Faculty of Science and Technology, University of Algarve, Campus de Gambelas, MeditBio, 8005-139 Faro, Portugal; ^3^Institute of Organic Chemistry with Centre of Phytochemistry, Acad. G. Bonchev Strl. Bl. 9, 1113 Sofia, Bulgaria; ^4^Department of Biological Sciences and Bioengineering, Faculty of Science and Technology, University of Algarve, Campus de Gambelas, 8005-139 Faro, Portugal; ^5^Department of Biological Sciences and Bioengineering, Faculty of Science and Technology, Center for Biomedical Research University of Algarve, Campus de Gambelas, 8005-139 Faro, Portugal

## Abstract

This study was performed to evaluate the total phenols, flavonoids, and antioxidant activities of twenty-four propolis samples from different regions of Morocco. In addition, two samples were screened regarding the antibacterial effect against four* Staphylococcus aureus *strains. Gas chromatography coupled to mass spectra (GC-MS) analysis was done for propolis samples used in antibacterial tests. The minimum inhibitory and minimum bactericidal concentration (MIC, MBC) were determined. The potential to acquire the resistance after sequential exposure of bacterial strains and the impact of adaptation to propolis on virulence using the* Galleria mellonella* were evaluated. Additionally, the effects of propolis extract on the bacterial adherence ability and its ability to inhibit the quorum sensing activity were also examined. Among the twenty-four extracts studied, the samples from Sefrou, Outat el Haj, and the two samples marketed in Morocco were the best for scavenging DPPH, ABTS, NO, peroxyl, and superoxide radicals as well as in scavenging of hydrogen peroxide. A strong correlation was found between the amounts of phenols, flavonoids, and antioxidant activities. Propolis extract at the MIC value (0.36 mg/mL) significantly reduced (*p* < 0.001) the virulence potential of* S. aureus* ATCC 6538 and the MRSA strains without leading to the development of resistance in the sequence of continuous exposure. It was able to impair the bacterial biofilm formation. The results have revealed that sample 1 reduces violacein production in a concentration dependent manner, indicating inhibition of quorum sensing. This extract has as main group of secondary metabolites flavonoids (31.9%), diterpenes (21.5%), and phenolic acid esters (16.5%).

## 1. Introduction

Reactive oxygen species (ROS) have a dual role in living beings. In adequate concentrations, they have a beneficial effect because they may constitute as defense against infectious agents, or being involved in some cellular signaling systems. Nevertheless, at higher concentrations, they damage cell structures (membranes) and biomolecules (lipids, nucleic acids, and proteins), leading to the known oxidative stress and consequently to the development of several age-dependent diseases (cancer, arteriosclerosis, arthritis, and neurodegenerative diseases, among others) [1]. The above shows that the organisms produce ROS, if not adequately controlled, through antioxidant systems; they are responsible for deleterious effects in the body. Such antioxidant systems may be endogenous [vitamin A, enzyme cofactors (Q10), nitrogen compounds (uric acid), and peptides (glutathione), enzymes (glutathione peroxidase, catalase, superoxide dismutase, glutathione reductase, and glucose-6-phosphate dehydrogenase)] or supplied through the diet (exogenous) [2]. Compounds present in food with potential antioxidant activity include vitamins C, E, and K, phenols (phenolic acids, flavonoids, thymol, and carvacrol, among others), and carotenoids [2, 3]. Thus, antioxidants, mainly those originating from natural products, are of a high important for the human health.

Elseways,* Staphylococcus aureus* (Gram positive bacterium) is both a human commensal and pathogen. This bacterium mainly colonizes the anterior nares of the nose; nevertheless the remaining part of the respiratory tract, as well as the gastrointestinal tract, skin, perineum, vagina, and axillae, may also be target of colonisation [4].* S. aureus* is one of the main causes of hospital-acquired infections, whenever the patients are submitted to intravenous drugs or under treatment with enteral feedings or dialysis, in postoperative surgical wounds in decubitus ulcers or in indwelling catheters, and almost always associated with a compromised defence mechanism [5–8]. In addition to the nosocomial infections, its incidence is increasing also in the community [9]. Moreover, this bacterium has a noteworthy potential to develop resistance to antibiotics. Methicillin resistant* S. aureus* (MRSA), which are resistant to *β*-lactam class of antibiotics (penicillins, cephalosporins, and carbapenems), are causing severe nosocomial infections [8, 10].

Equally important to mention that, bacterial pathogenic cells that adhere closely to an abiotic or biotic surface are responsible for serious infections, because such biofilm matrix and phenotypic characteristics of the bacteria confer resistance to the action of antimicrobial agents. Furthermore, such biofilm may develop either on living tissues or on inert surfaces of medical devices, leading to infections in urogenital, gastrointestinal, respiratory tracts and eyes, and wounds generally difficult to treat [11, 12].

Besides, intercellular communication within biofilms has been attributed to the quorum sensing system (QS) which is mediated by diffusible small signalling molecules [12, 13]. Bacteria in biofilms are resistant to disinfectants and antibiotics leading to persistent infections, due to their higher virulence. To find compounds which are able to interfere with the bacterial QS system will regulate the virulence of bacterial pathogens, regardless of multidrug resistance [13].

Propolis, a natural resinous substance constituted by resin (50%), wax (30%), essential oils (10%), pollen (5%), and other substances (5%), such as debris, minerals, and organic compounds, possess antimicrobial, anti-inflammatory, antioxidant, antidiabetic, spasmolytic, anaesthetic, anticancer, and immunomodulatory effects [14].

The antioxidant activity of propolis extracts and/or their constituents has been deeply evaluated worldwide, including in the Mediterranean basin: Algeria [15–17]; Croatia [18, 19]; Cyprus [20]; Egypt [21, 22]; Greece [20]; Italy [23–25]; Macedonia [26]; Serbia [27]; Slovenia [28]; Spain [29, 30]; and Turkey [31, 32], using several methodologies: antioxidant; reducing power; chelating activity; capacity for scavenging the DPPH, ABTS, peroxyl [oxygen radical absorbance capacity (ORAC)] and NO free radicals; capacity for scavenging the superoxide anion radical; and capacity for scavenging hydrogen peroxide. Very few works on antioxidant activity of Moroccan propolis were found [33, 34]. Otherwise, the* in vitro* antimicrobial activity of propolis against* S. aureus* and MRSA strains has been reported [15, 35–44]; however in Moroccan propolis such information is scarce or even absent.

The aim of the present work was to evaluate propolis as a natural product regarding the antioxidant activity, measured through the capacity for scavenging diverse free radicals (DPPH, ABTS, superoxide, NO, and peroxyl); capacity for reducing ion metals or chelating activity of hydro-alcoholic extracts of propolis was assessed, allowing to determine by which mechanisms they act as antioxidants, at the same time, the* in vitro* antibacterial activity of hydro-alcoholic extracts of Moroccan propolis against* S. aureus* strains, mainly MRSA clinical isolates, as well as the effect of the adaptation of the* S. aureus* strains on the susceptibility to propolis extract, and its influence on the adherence of those microorganisms to surfaces. The capacity for preventing this adherence reveals to be of particular interest because the adherent bacterial cells (biofilm bacteria) are more resistant to antibiotics than planktonic cells. In addition the ability of propolis extract to inhibit the QS system, diminishing the virulence of bacterial pathogens, was also screened.

## 2. Methods

### 2.1. Chemical

Iron (II) chloride anhydrous and 6-hydroxy-2,5,7,8-tetramethylchroman-2-carboxylic acid (Trolox®) were purchased from Fluka Chemicals (Buchs, Switzerland). Thiobarbituric acid, ferulic acid, DNP (2,4-dinitrophenylhydrazine), quercetin, sulphuric acid (H_2_SO_4_), potassium hydroxide (KOH), ABTS (2,2'-azino bis(3thylbenzothiazoline-6-sulphonic acid), fluorescein sodium salt, sodium nitrite ASC reagent, and eryodictiol were purchased from Fluka Biochemika, Sigma-Aldrich, Steinheim, Germany. Acetic acid, potassium dihydrogen phosphate (KH_2_PO_4_), dipotassium hydrogen phosphate (K_2_HPO_4_), and trichloroacetic acid were purchased from VWR, Leuven, Belgium. Phenazine methosulfate (PMS), nicotinamide adenine dinucleotide disodium salt hydrate (NADH), ferrozine iron reagent hydrate; AAPH [2,2'-Azobis (2-methylpropionamidine) dihydrochloride]; ethylenediaminetetraacetic acid (EDTA), and potassium persulfate were purchased from Acros organics, New Jersey, USA. Nitrotetrazolium blue chloride and trichloroacetic were purchased from Sigma Aldrich Chemie, Steinheim, Germany. Phosphate buffered saline tablets (PBS) were from Fisher Scientific, New Jersey, USA. Sodium nitroprussiate dehydrate, 2,2'-diphenyl-1-picrylhydrazyl (DPPH), and potassium hexacyanoferrate were from Riedel-de Haën, Sigma-Aldrich, Seelze, Germany. Griess reagent system was purchased from Promega Corporation, Madison, USA. Calcium chloride, Folin Ciocalteu's phenol reagent, AlCl_3_, di-potassium hydrogen phosphate anhydrous (K_2_HPO_4_), and hydrogen peroxide (H_2_O_2_) were purchased from Panreac Quimica, Montcada i Reixac, Barcelona, Spain. Potassium dihydrogen phosphate (KH_2_PO_4_) and Na_2_CO_3_ were purchased from Riedel de Haen (Seelze, Germany, Riedel-de-Haën Laboratory Chemicals, Germany). Potassium chloride (KCl) was purchased from Analar Normapur, Geldenaaksebaan, Leuven, Belgium. Ammonium heptamolybdate tetrahydrate PA was purchased from PRONALAB, LISBOA, Portugal. The medium brain-heart infusion (BHI), Luria-Bertani (LB), and bacteriological agar type E were purchased from Biokar Diagnostics (Beauvais, France). Crystal violet was from Merck, Germany.

### 2.2. Propolis Extraction

Hydro-alcoholic extracts of twenty-four propolis samples, originating from different location in Morocco (Table 1), were obtained, as described by El-Guendouz et al. [45], with slight modification. One gram of each propolis sample was chopped into small pieces and extracted by maceration using 30 mL of 70% ethanol and maintained for one week at 37°C under agitation (200 rpm). The resulting solution was filtered under vacuum. A clear solution, without further purification, was used for successive analyses. First of all, polyphenol contents and antioxidant activities were screened for all samples (twenty-four) while only two samples were used for microbiological study (one among the most active regarding the antioxidant activity and polyphenol contents and the second among the weaker samples regarding the same parameters).

### 2.3. Determination of Different Phenolic Groups

#### 2.3.1. Total Phenol Content

The total polyphenol content in propolis samples was determined using the method of [46]. Hydro-alcoholic extracts (25 *μ*L) were mixed with 125 *μ*L of Folin-Ciocalteu reagent (0.2 N) and 100 *μ*L of 7.5% Na_2_CO_3_, and the absorbance was measured at 760 nm after 2 h of incubation at room temperature. The total polyphenol content was expressed as mg per g of Ferulic Acid equivalents using a calibration curve.

#### 2.3.2. Total Flavones and Flavonol Content

The amounts of flavones and flavonols in extracts were determined according to the method [47], with minor modification. An amount of 100 *μ*L of Al_2_Cl_3_ (20%) was added to 100 *μ*L of extract, and after one hour at room temperature the absorbance was measured at 420 nm. Total flavones and the flavonols content were calculated as quercetin equivalents (mg per g) using a calibration curve.

#### 2.3.3. Total Flavanones and Dihydroflavonol Contents

The total quantification of flavanone and dihydroflavonol compounds was performed as described by some authors [48]. Briefly, 75 *µ*L of sample or standard and two milliliters of DNP solution (one gram DNP in two milliliters 96% sulfuric acid, diluted to 100 mL with methanol) were heated at 50°C for 50 min. After cooling at room temperature, the mixture was diluted to 10 mL with 10% KOH in methanol (w/v). A sample of one milliliter of the resulting solution was added to 10 mL methanol and diluted to 50 mL with methanol. Absorbance was measured at 486 nm.

### 2.4. Antioxidant Activity

#### 2.4.1. DPPH Free Radical Scavenging Activity

Scavenging of the DPPH radical was assayed following the method of [49], with some modification. Solutions with different extract concentrations were prepared and 25 *µ*L of each solution was added to 150 *µ*L of DPPH solution (63.4 *µ*M) and 125 *µ*L of 96% ethanol. Absorbance measurements were read at 517 nm, after 30 min of incubation time at room temperature. Reduction of the amount of the DPPH radical present was measured using a decrease in the absorption value at 517 nm. The extract concentration providing 50% inhibition (IC_50_) was calculated using a graph of the scavenging effect percentage against the extract concentration. The scavenging effect percentage was calculated from the formula: [(A_0_-A_1_/A_0_) x 100], where A_0_ is the absorbance of a negative control (blank sample containing the same amount of solvent of extraction and DPPH solution) and A_1_ is the absorbance of the sample. The percentage was plotted against the extract concentration, and IC_50_ values were determined (concentration of extract able to scavenger 50% of the DPPH free radical).

#### 2.4.2. ABTS Free Radical-Scavenging Activity

Determination of the ABTS radical scavenging activity was carried out as reported by [50]. Briefly, the ABTS radical was generated by reaction of a 7 mM ABTS aqueous solution with K_2_S_2_O_8_ (2.45 mM) in the dark for 16 h and adjusting the absorbance at 734 nm to 0.7 at room temperature. Samples (25 *μ*L) were added to 275 *μ*L of ABTS and the absorbance at 734 nm was read after 6 min. The capability to scavenge the ABTS^+^ was calculated using the formula: ABTS scavenging activity (%) = [(A_0_-A_1_)/A_0_] × 100 (%), where A_0_ is the absorbance of the control (ethanol 70% instead sample) and A_1_ is the absorbance in the presence of the sample. The sample concentration providing 50% inhibition (IC_50_) was obtained by plotting the inhibition percentage against extracts concentrations.

#### 2.4.3. Evaluation of Total Antioxidant Capacity by Phosphomolybdenum

The antioxidant activities of samples were evaluated by the phosphomolybdenum method as described by [51] and expressed relative to that of ascorbic acid. Briefly, an aliquot of 50 *µ*L of propolis sample was mixed with one milliliter of the reagent solution (0.6 M sulfuric acid, 28 mM sodium phosphate, and 4 mM ammonium molybdate). Ethanol 70% was used as blank instead of propolis solution. The reaction mixture was vortex-mixed and let to stand in a water bath at 95°C for 90 min. The tubes were cooled to room temperature and the absorbance was measured at 695 nm. The experiment was conducted in triplicate and values are expressed as mg equivalents of ascorbic acid per gram of propolis.

#### 2.4.4. Nitric Oxide Scavenging Activity

The nitric oxide (NO) scavenging activity was measured according to [52]. In this method 50 *µ*L of different concentration of sample was added to 50 *µ*L of 10 mM sodium nitroprusside in PBS into a 96-well plate and the plate was incubated at room temperature for 90 min. Finally, an equal volume of Griess reagent was added to each well and the absorbance was read at 532 nm. Several concentrations of samples were made and the percentage inhibition calculated from the formula [1 - (A_sample_ - A_sample  blank_)/(A_control_ - A_control  blank_)]x100, where (A_sample_ - A_sample  blank_) is the difference in the absorbance of a sample, with or without 10 mM sodium nitroprusside, and (A_control_ - A_control  blank_) is the difference in the absorbance of the PBS control, with or without 10 mM sodium nitroprusside. The sample concentration providing 50% inhibition (IC_50_) was obtained by plotting the inhibition percentage against extracts concentrations.

#### 2.4.5. Chelating Metal Ions

The degree of chelating of ferrous ions by the propolis samples was evaluated according to [53]. Briefly, 0.1 mL of different concentration of samples was incubated with 0.05 mL of FeCl_2_.4H_2_O (2 mM). The addition of 0.2 mL of 5 mM ferrozine initiated the reaction, and after10 min, the absorbance at 562 nm was measured. An untreated sample served as the control. The percentage of chelating ability was determined according to the following formula: [(A_0_-A_1_)/A_0_ x100], in which A_0_ is the absorbance of the control and A_1_ is the absorbance of propolis sample. The values of IC_50_ were determined as reported above.

#### 2.4.6. Scavenging Ability of Superoxide Anion Radical

Scavenging ability of superoxide anion radical was evaluated as previously reported by [54]. Superoxide anions were generated in a nonenzymatic PMS-NADH system by oxidation of NADH and assayed by reduction of nitroblue tetrazolium (NBT). The superoxide anion was generated in 300 *µ*L of phosphate buffer (19 mM, pH=7.4) containing NBT (43 *µ*M) solution, NADH (166 *µ*M) solution, and different concentrations of propolis. The reaction was started with the addition of PMS solution (2.7 *µ*M) to the mixture. The reaction mixture was incubated at room temperature for 10 min and the absorbance reading was performed at 560 nm in a microplate reader (Tecan Infinite M200, Tecan, Austria). The percentage of inhibition was calculated using the following equation:

(1) $$\begin{array}{c} Inhibition=\left[\;\frac{\left(A_{0}\text{-}A_{1}\right)}{A_{0}}\;\right]x100\left(\;\%\;\right) \end{array}$$

where A_0_ is the absorbance of the control (without sample) and *A*_1_ is the absorbance in the presence of the sample. Tests were carried out in triplicate. The sample concentration providing 50% inhibition (IC_50_) was obtained by plotting the inhibition percentage against extracts concentrations.

#### 2.4.7. Hydrogen Peroxide Scavenging Capacity

The ability of the propolis extracts to scavenge hydrogen peroxide was determined according to the method of [55], with slight modification. A solution of hydrogen peroxide (40 mM) was prepared in phosphate buffer (50 mM, pH=7.4). Fifty *μ*L of each propolis concentration was added to a hydrogen peroxide solution (0.6 mL, 40mM), one milliliter of phosphate buffer (50 mM, pH=7.4), and 1650 *µ*L of distilled water. Absorbance of hydrogen peroxide at 230 nm was determined 20 minutes later against a blank solution containing the phosphate buffer without hydrogen peroxide. Tests were carried out in triplicate and the values of IC_50_ were determined as reported above.

#### 2.4.8. Scavenging Ability of Peroxyl Radicals by the Oxygen Radical Absorbance Capacity (ORAC)

An ORAC assay is based on the capacity of antioxidants in a sample to quench peroxyl radicals generated from thermal decomposition of AAPH. The ORAC method used fluorescein (FL) as the fluorescent probe following the method of [56]. An amount of 0.414 g of AAPH was dissolved in 10 mL of phosphate buffer 75 mM (pH 7.4) and kept in an ice bath. A solution of fluorescein (0.00419 mM) was prepared in phosphate buffer and kept in the dark at 4°C. A new concentration (8.16×10^−5^ mM) was prepared before reactions. A Trolox standard (0.02 M) was prepared in phosphate buffer and diluted to 50, 25, 12.5, and 6.25 *µ*M. As the ORAC assay is extremely sensitive, the samples must be diluted (1/1,000; 1/10,000) before analysis to avoid interference. An amount of 150 *µ*L of a fluorescein working solution, 25 *µ*L of sample, and either a blank (Milli-Q water) or a standard were mixed on a plate, which was then covered with a lid and incubated in a preheated (37°C) microplate reader for 10 min. An amount of 25 *µ*L of AAPH solution was then added to each well. The microplate was shaken for 10 s and then the fluorescence was read every minute for two hours at an excitation wavelength of 485 nm and an emission wavelength of 527 nm. The net area under the curve (AUC) of the standards and samples was calculated. A standard curve was obtained by plotting Trolox concentrations against the average net AUC of two measurements for each concentration. Final ORAC values were calculated using a regression equation between the Trolox concentration and the net AUC and are expressed as *µ*mol of Trolox/g propolis.

#### 2.4.9. Reducing Power Assay

The reducing power was determined according to the method described by [57], with slight modifications. The extracts (50 *μ*L) were mixed with 500 *μ*L of 0.2M sodium phosphate buffer (pH=6) and 500 *μ*L of 1% potassium ferricyanide (K_3_F_e_CN_6_). The mixture was incubated at 50°C for 20 min. After 500 *μ*L of 10% trichloroacetic acid was added, the mixture was centrifuged for 10 min at 3000 rpm. 500 *μ*L of the supernatant liquid was mixed with 500 *μ*L of distilled water and 100 *μ*L of 0.1% ferric chloride. The absorbance of the mixture was measured at 700 nm.

### 2.5. Antimicrobial Activity

After the screening of polyphenol content and the antioxidant activities of all propolis samples, only two samples were chosen for microbiological test (samples 1 and 7) based on the previous test performed.

#### 2.5.1. GC-MS Analysis of Propolis Extract

GC-MS analysis was done for the two samples (1 and 7) chosen for the antimicrobial assays. The analysis was performed with a Hewlett–Packard gas chromatograph 5890 series II Plus linked to a Hewlett–Packard 5972 mass spectrometer system equipped with a 30 m long, 0.25 mm i.d., and 0.5 *µ*m film thickness HP5-MS capillary column. The work conditions were the same as previously reported [58]. Semi-quantification was carried out by internal normalisation with the area of each compound. The addition of individual areas of the compounds corresponds to 100% area. Compound identification was performed using commercial libraries and comparison of mass spectra and retention times of reference compounds.

#### 2.5.2. Bacterial Strains and Growth Conditions

The bacterial strains were cultivated in BHI. For solid media, agar was added at 1.5 %, w/v. The bacterial strains of* S. aureus* used in this study are indicated in Table 5. Bacteria were stored in BHI supplemented with 25 % (v/v) glycerol at -80°C. Prior to use, bacteria were transferred to fresh BHI agar plates and incubated at 37°C.* Chromobacterium violaceum* CV026 (HgR, cvil:Tn5 xylE, KanR and spontaneous resistance StrR) was a gift from Professor Mondher EL Jaziri, Plant Biotechnology Laboratory, Université Libre de Bruxelles, Belgium.

#### 2.5.3. Disc Diffusion Method

A paper disc diffusion assay to assess the preliminary antibacterial activity of two propolis samples (most active (1) and the less active (7) samples regarding their total phenol content) was performed according to [59].* S. aureus* cultures were incubated overnight before the OD_600_ was adjusted to 0.3-0.4. Hundred microliters of this bacterial culture were then spread on BHI agar plates and left to dry at room temperature for a few minutes. Then sterile filter paper discs were placed over the plate and propolis extracts at concentration of 0.24, 0.49, 0.73, and 0.98 mg/mL were loaded onto the discs. Diluted ethanol 70% was used as a negative control and the chloramphenicol disc (30 *μ*g/mL) as a positive control. The plates were incubated at 37°C for 24 hours. After the incubation time the inhibition zones were determined. Three biological and three technical replicates (N=9) were done.

#### 2.5.4. Determination of the Minimum Inhibitory Concentration

The minimum inhibitory concentration (MIC) of propolis samples (1 and 7) was determined using a microdilution method according to [60]. Concentrations of propolis were 0.24; 0.36; 0.49; 0.61; 0.73; 0.98; and 1.22 mg/mL. Each* S. aureus* strain was cultivated in BHI agar plates for 24 h at 37°C. A loop of isolated bacterial colonies from each plate was inoculated into 10 mL of BHI broth, and the culture was incubated overnight in a shaking water bath at 37°C, to prepare a suspension with a turbidity of 0.5 of the McFarland scale (10^8^ CFU/mL). 100 *µ*L of the overnight bacterial culture was centrifuged, and the pellet was resuspended with the same volume of medium supplemented with the appropriate propolis concentration, then 20 *µ*L of the bacterial suspension was inoculated into each well of a flat-bottom microplate (Greiner, Labortechnik, Frickenhausen, Germany) previously filled with 180 *µ*L of BHI supplemented with propolis at the proper concentration. Wells containing the culture medium supplemented with ethanol 70% or chloramphenicol (30 *µ*g/mL) were included, as control. Negative control was constituted by the culture in BHI and no propolis added. Three biological and three technical replicates for each strain were used (N = 9). The growth was followed by spectrophotometry (OD) in a microplate reader (Tecan Infinite M200, Tecan, Austria) at optical density of 600 nm. The MIC value was considered as the lowest concentration of propolis that inhibits the visible bacterial growth (no increase in the OD after 24–48 h). The lowest concentration that did not allowed the recovery of cells in agar plates was considered the minimum bactericidal concentration (MBC).

#### 2.5.5. Effect of the Adaptation of S. aureus Strains on the Susceptibility to Propolis Extract and Antibiotics

According to the disc diffusion and MIC value results the remaining microbiological tests will be done only with sample 1. Adaptation procedure (successive passage) was performed as described by [60]. The tested concentrations of propolis (sample 1) were 0.12; 0.24; 0.36; 0.49; and 0.61 mg/mL. Each bacterial strain was cultivated on BHI agar plates at 37°C during 24 h. 10 mL of BHI broth was inoculated with a loop of isolated colonies from each plate, and the culture incubated overnight. 300 *µ*L of the overnight bacterial culture was centrifuged (2,790 x* g*, 5 min at 4°c) and the pellet was resuspended in 300 *µ*L BHI supplemented with 0.12 mg/mL of propolis extract and then inoculated into 10 mL of BHI with the same concentration of propolis extract. The bacterial culture was incubated for 24 h in a shaking water bath at 37°C and then was transferred sequentially at the same concentration of propolis extract for 4 days. The bacterial growth at each successive passage was followed by spectrophotometry at OD_600_ nm. Following the four days of sequential passages, bacteria were transferred to BHI with higher concentration of propolis extract. Agar disc diffusion was performed each day of the sequential passages of each concentration. The purpose was to evaluate the bacteria susceptibility to antibiotics and eventual development of antibiotic resistance (gentamicin, erythromycin, chloramphenicol, and tetracycline). These procedures were repeated until bacteria cease to grow. Culture with no propolis extract was used as control in parallel. Three independent replicates were performed.

#### 2.5.6. Influence of Propolis Extract on Adherence

The influence of propolis extract (sample 1) on adherence of* S. aureus* was performed according to [61] with slight modifications. Briefly, 200 *µ*L of an overnight culture (OD = 0.4-0.5), prepared as described above, was centrifuged and the pellet was resuspended with medium supplemented with propolis extract at the MIC value (0.36 mg/mL), chlorhexidine 0.2% (positive control), and BHI only (negative control). The prepared culture was inoculated into each well of a flat-bottom microplate and left to adhere for 30 min at room temperature in a flow cabinet. After incubation nonadherent cells and residues of media component were removed by washing the wells with sterile PBS. Wells were air dried for 15 min. The adherent cells were heat-fixed at 80°C for 30 min, stained with 0.1% crystal violet, and kept for 10 min to react. The wells were washed again before the dissolution of the stain by addition of ethanol–acetone (80:20), and the OD was determined with a microplate reader at 595 nm.

#### 2.5.7. Screening of Propolis Extract for Anti-QS Properties

This assay was performed as described by [62] with some modifications.* Chromobacterium violaceum* CV026 was cultured on LB agar plates at 30°C during 24 h. LB broth was inoculated from each plate, and the culture was incubated overnight in a water bath at 30°C. Eight mL of the overnight culture was adjusted to OD_600_ of 1.2 and transferred into 40 mL of warm molten LB agar, followed by the addition of* N*-hexanoylhomoserine lactone (C6-HSL) at final concentration of 0.12 *µ*g/mL. The agar mixture was mixed and poured into the Petri dish and left to solidify inside a flow cabinet for one hour. Small wells were made on the solidified agar plate with the bottom of a Pasteur pipette. Wells were filled with 40 *µ*L of propolis extract at the concentration 0.24; 0.36; 0.49; 0.61; 0.73; 0.98; and 1.22 mg/mL. The plate was incubated for 24 hours at 30°C to check for the violacein inhibition. The positive control for the QS inhibition in this assay was (+)-catechin and ethanol 70% diluted was used as the negative control.

#### 2.5.8. Influence of Propolis Extract on Virulence

The effect of adaptation of propolis (sample 1) on virulence of* S. aureus* strains was determined using larvae of the Greater Wax Moth* Galleria mellonella* (Lepidoptera: Pyralidae) according to [63]. The insects weighing between 250 and 350 mg were reared during the first and second instars with pollen and wax then with an artificial diet containing a mixture of glycerol, honey, water, dry dog food, and wheat bran. It is noted that the development of the insects was carried out in the dark at 30°C. * S. aureus* strains were previously adapted to subinhibitory propolis concentration (0.24 mg/mL) for three successive passages. Each* S. aureus* culture (10^8^ CFU mL^−1^) was centrifuged at 2,790 x* g* for 5 min at 4°C, then the bacterial cells were resuspended in one milliliter of sterile PBS. First larvae were superficially disinfected with ethanol at 70% v/v and then were infected with 10 *μ*L of the initial suspension (10^6^ CFU) by injection on the second right proleg using a 50 *μ*L microsyringe. Larvae were placed in sterile Petri dishes at 37°C and death checked every 24 h for 5 days. Death was considered when no response to touch was observed. Three independent experiments were carried out. PBS was injected to the insect control.

### 2.6. Statistical Analysis

Data were analysed by ANOVA using the SPSS 23.0 program (Inc., Chicago, IL, USA). Statistical significance was set as* p*< 0.05; when the analysis was statistically significant, Tukey's post hoc test was done. The survival data were treated using GraphPad Prism 5 statistical software.

## 3. Results and Discussion

### 3.1. Phenols

Table 1 depicts the phenol content of Moroccan propolis samples. Samples 17, 23, and 1 had the highest concentrations of total phenols (265.37, 264.80, and 205.82 mg ferulic acid equivalent (FAE)/g, respectively), total flavones and flavonols (129.60, 80.97, and 53.50 mg QE/g, respectively), and total dihydroflavonols and flavanones (6.67, 15.95, and 7.69 mg EE/g, respectively). Among these samples, propolis extract 1 had the lowest concentrations of total phenols, flavones, and flavonols, whereas propolis extract 17 had the lowest concentration of dihydroflavonols and flavanones (Table 1). Shortly, samples 17 and 23 had similar amounts of total phenols, but sample 17 had higher flavones and flavonols and lower concentrations of flavanones and dihydroflavonols than sample 1. The polyphenol content of these samples is superior to those described by [47]. In this case, 91.22 mg caffeic acid equivalent (CAE)/g was the best value found among the 14 samples of propolis collected at different places of Morocco. With the exceptions of samples 24, 21, 7, and 12 with the lowest concentrations of total polyphenols (7.06-9.53 mg FAE/g) and those with the highest concentrations of the same metabolites, the remaining samples had similar amounts of phenols to those reported for the neighbor country, Algeria, 10–47 mg gallic acid (GA)/g [17], and superior to the majority of samples for Morocco (0.7–65.7 mg CAE/g) [47]. Lower amounts of flavonoids were also reported by some authors [17, 47]. When compared to the results presented in the present work, the highest concentration of total flavonoids detected by these authors did not surpass 35 mg QE/g for Moroccan propolis or 30 mg QE/g for Algerian propolis.

### 3.2. Antioxidant Activity

**Figure 1:**
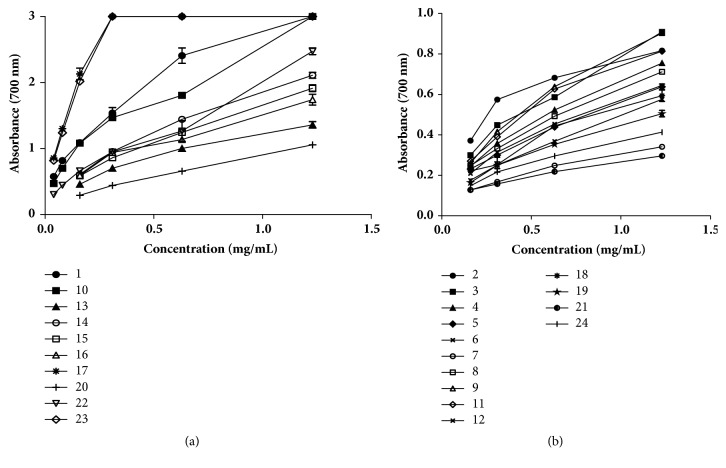
The reducing power of the hydro-alcoholic extracts of propolis from different areas of Morocco. (a) Group of samples with the best reducing power; (b) group of samples with the lower reducing power.

The antioxidant activity was checked through diverse methods: capacity for scavenging diverse free radicals (DPPH, ABTS, superoxide, NO, peroxyl), or chelating activity. The results are presented in Table 2. The capacity for reducing ion metals is depicted in Figure 1.

The results will be only compared with those from Mediterranean origin found in articles listed in the ISI Web of Knowledge [15–17, 27, 28, 31, 34, 47, 50, 64–70].

Among all propolis extracts, sample 1 had the best total antioxidant activity (48.95 meq Ascorbic Acid Equivalent /g) (Table 2), higher than those already reported for Moroccan propolis [34]. This sample, along with samples 10, 17, and 23, presented the lowest IC_50_ values for the assays that measure the ability for scavenging ABTS and DPPH free radicals, that is, those that showed the best capacity for scavenging such free radicals. Samples 13-16 also had significant higher capacity for scavenging the ABTS free radicals (Table 2).

In the DPPH and ABTS assays, the lowest IC_50_ values (0.007–0.024 mg/mL and 0.002–0.044 mg/mL, respectively) which correspond to the best activities are within the range found for propolis samples from Morocco [47]. For the DPPH method, Algerian propolis extracts had lower activity (IC_50_ values ranging from 32.3 to 600 *μ*g/mL) [15]. In the same work, these authors reported that the purified compounds had higher activity than the extracts, being kaempferol and phenetyl-(*E*)-caffeate those possessing the best activities, with IC_50_ of 4.2 and 9.9 *μ*g/mL, respectively. The same authors found a direct correlation between the amount of phenols, including flavonoids, and the capacity for scavenging the free radicals. For scavenging about 60% of DPPH free radicals, [17] reported that it was necessary at least 100 *μ*g/mL of ethanolic extract of propolis from Algeria. Like [15], Benhanifia et al. [17] also found a remarkable correlation between phenol content and antioxidant activity, measured through the capacity for scavenging DPPH free radicals, but absent in the capacity for reducing ferric ions. The authors [16] found as IC_50_ value, 25 *μ*g/mL, for an ethanolic extract of propolis from Algeria, closer to those reported in the present work.

When the antioxidant activity was evaluated through the capacity for scavenging ABTS free radicals, beyond those samples with the highest activity for scavenging DPPH free radicals (1, 10, 17, and 23), other ones were equally good scavengers of ABTS, such as 13-16 (Table 2). These samples did not possess as high amounts of phenols or flavonoids as the remaining samples. These results reveal the importance of some compounds and/or their functional groups present in the extracts on the activity and that show behaviour somehow different in the presence of DPPH or ABTS free radicals [64, 65].

The capacity for scavenging DPPH and ABTS, cheap and simple methods, can give information about the antiradical activity of samples; nevertheless they are not natural radicals, whereby the evaluation of the capacity of samples for scavenging ROS produced by the organisms is with a high interest. The most important reactive molecules include superoxide and hydrogen peroxide.

Samples 10 (IC_50_ = 0.253 mg/mL) and 23 (IC_50_ = 0.213 mg/mL) had the best capacity for scavenging superoxide radical anions, poorer when compared to the best IC_50_ values found for samples from Morocco (IC_50_ = 0.15 mg/mL), previously reported [34], and markedly poorer to those reported by Gülçin et al. (2010) for propolis samples from Turkey [31], with IC_50_ = 9.89 *μ*g/mL. Mavri et al. [28] also evaluated the capacity of Slovenian propolis for scavenging superoxide anion radicals, showing that 0.02 mg/mL of propolis phenols present in the extracts were able to scavenge between 6.8 and 21% of superoxide anion radicals, depending on these percentages of the ethanol: water ratio of the extraction solvent.

The capacity for scavenging hydrogen peroxide was significantly higher for samples 1 (IC_50_ = 0.086 mg/mL) and 23 (IC_50_ = 0.048 mg/mL), being such values about tenfold higher than those reported by [31] for Turkish samples (6.54 *μ*g/mL), and therefore with poorer ability for scavenging H_2_O_2_. Potkonjak et al. [27] determined the antioxidant activity of commercial propolis extracts from Serbia through direct current polarography, expressed in H_2_O_2_ equivalent (the volume of H_2_O_2_ that corresponds to 1.0 mmol/L H_2_O_2_ decrease). The values found by the authors ranged from 1.71 to 8.00 *μ*L. Once the unities are different, it is not possible to compare the results obtained by [27] with the values obtained in the present work.

The capacity of samples 1, 10, and 23 for scavenging superoxide and hydrogen peroxide not only prevents the generation of more reactive species, such as hydroxyl radicals, but also avoids the interaction between ROS and biomolecules [66].

The best capacity for scavenging peroxyl free radicals, measured through the method called ORAC, that measures the capacity for scavenging peroxyl radicals, was detected for samples 17 (1723.289 *μ*mol TE/g) and 23 (1616.654 *μ*mol TE/g), inferior to the maximal observed for Moroccan propolis [47]. The best antiradical capacity of those samples (17 and 23) is revealed to be important since they may prevent the propagation of lipid peroxidation caused by the peroxyl radicals [67].

Only sample 17 was significantly better as NO scavenger (IC_50_ = 0.025 mg/ml) when compared to the remaining samples, whilst sample 13 presented the best chelating activity (IC_50_ = 0.251 mg/ml) (Table 2). The capacity for scavenging NO radicals was better than those found by [34] for Moroccan propolis (IC_50_ = 0.08-2.90 mg/mL). The best chelating activity found in the present work was inferior to that previously reported (IC_50_ = 0.13 mg/mL) also for Moroccan propolis [34] and significantly inferior to those found for Turkish samples (0.012 mg/mL) [31], but better when compared to the Slovenian propolis (2 mg/mL) [28].

The capacity of sample 17 for nitric oxide quenching can avoid the generation of peroxynitrite, in the presence of superoxide anion radicals, that irreversibly binds to proteins impairing cellular function [68].

The presence of iron ions and hydrogen peroxide may trigger the formation of the most reactive hydroxyl radicals, through the Fenton reaction; therefore the absence of these ions as well as other transition metal ions, such as copper, is important. Sample 13 is revealed to be the most active in what concerns the capacity for chelating iron ions [68].

Two main groups may be considered in terms of capacity for reducing Fe^3+^ to Fe^2+^ by propolis extracts (Figures 1(a) and 1(b)). Samples 1, 10, 13, 14, 15, 16, 17, 20, 22, and 23 are in group one (Figure 1(a)), being those with the best reducing capacity (for the higher concentration used, the absorbance was ≥ 1, at *λ*=700 nm); and the remaining samples belong to group two, that is, those with significant lower reducing activities (Figure 1(b)). It is noteworthy to refer that with the exception of samples 20 and 22, which were introduced in the first group, the remaining samples belonging to the same group are also those with the best capacity for scavenging ABTS free radicals. Such may partly explained by the mechanism involved in the reaction between ABTS free radicals and compounds, which is predominantly based in single electron transfer mechanism, that is, the same mechanism that occurred during the reduction of Fe^3+^ to Fe^2+^. At least two factors may have contributed to the lower number of samples (1, 10, 17, and 23) with relative high capacity for scavenging DPPH free radicals when compared to the number of samples with significant higher capacity for scavenging ABTS free radicals: (a) the mechanisms involved in the reaction between DPPH and compounds are a mix of single electron transfer and hydrogen atom transfer mechanisms; and (b) the reaction between extracts, constituted by several compounds, and DPPH is less than the total reactivity of individual compounds [65].

A negative correlation between IC_50_ values (DPPH, ABTS, NO, and superoxide) and phenol content (*p*<0.01) was found, which means higher activity with higher concentrations of phenols. The same correlation was observed between the same values and the amounts of flavonoids (flavanone, di-hydroflavonols, flavones, and flavonols) (Table 3). A positive correlation was observed between the values for ORAC test and the concentrations of phenols, independent on the type, meaning an increase of the capacity for scavenging free peroxyl radicals and phenols. The same was observed between the amounts of phenols and the total antioxidant activity determined by the molybdate method, although there is higher correlation between the values and the amounts of flavanones and dihydroflavonols (Table 3). A positive correlation between IC_50_ values and chelating activity was found. In this case, it seems that phenols, independent on the type, have a negative effect on the chelating activity, impairing it. The chelating efficiency of phenols on Fe^2+^ depends on the number of hydroxyl groups on the benzene ring as well as on the hydroxyl substitution in the* ortho* position [69]. An absence of correlation between chelating activity and phenol amounts was already reported by [50] for commercial honeys for Morocco. Although the presence of high levels of polyphenol in cocoa extracts, Andújar et al. [69] also found an absence of ability of samples for chelating Fe^2+^ ions. The results found in the present work suggest that the observed chelating activity can be assigned to compounds other than phenols.

### 3.3. Antimicrobial Properties

#### 3.3.1. Antistaphylococcal Activity of Propolis

The results of the antibacterial activity of propolis extracts against* S. aureus* strains are indicated in Table 4. Our results showed a visible inhibition zone, particularly sample 1, at all concentrations tested against the four strains. The highest inhibition zone (27.00 ± 1.52 mm; 25.67 ± 0.57 mm; 31.67 ± 1.73 mm and 32.33 ± 1.51 mm) was recorded from sample 1 at 0.98 mg/mL, higher than chloramphenicol (26.00 ± 2.51 mm; 19.00 ± 0.57mm; 24.33 ± 0.01mm and 24.67 ± 2.29mm) for MRSA strains, while sample 7 showed a lower inhibition zone (14.33±0.57 mm; 16.33±4.93 mm; 16.00±1.01 mm; and 17.67±2.88 mm) at the same concentrations. At concentration of 0.24 mg/mL and 0.49 mg/mL, the inhibition zones were very small <20mm for sample 1 and <10 mm for sample 7 (data not shown). Ethanol (negative control) did not show antibacterial activity, as expected. The diameters of inhibition zones were increased with respect to the volume of the extracts.

The best activity found for sample 1 was confirmed through the values of MIC and MBC found. Propolis sample 1 showed a MIC value of 0.36 mg/mL and the MBC ranged from 0.98 to 1.22 mg/mL for* S. aureus* tested strains.

No differences were observed between the MIC values of sample 1 (0.36 mg/mL) for all strains tested. However, the MBC values determined for* S. aureus* MRSA2, MRSA15, and MRSA16 were 1.22 mg/mL, except for* S. aureus* ATCC 6538, which was 0.98 mg/mL. In contrast, the susceptibility of* S. aureus* strains to sample 7 was not proved. The concentrations tested did not affect the bacterial growth.

**Figure 2:**
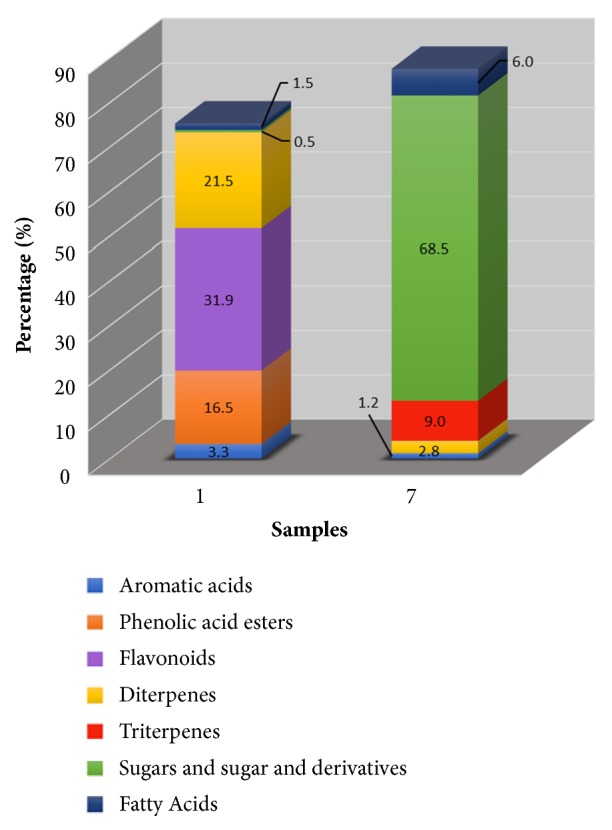
Group of compounds present in propolis samples S1 and S7.

The differences found in the activities between samples 1 and 7 may result for their distinct chemical composition. In Figure 2, it is possible to see that sample 7 was predominantly constituted by sugars and their derivatives (68.5%), whereas sample 1 had as main group of secondary metabolites, the flavonoids, diterpenes, and phenolic acid esters [45]. The high percentage of sugars is mainly constituted by monosaccharides (52.6%), which may reveal appreciable quantities of honey and therefore an inadequate harvesting of propolis.

Sample 7 had also triterpenes, particularly sterols represented particularly by lanosterol lupeol (3.5%), (3-*β*-OH) (2.9%), lanosterol (3-*α*-OH) (1.2%), and *α*-amyrin (0.4%). The diterpenes were the third abundant group in the propolis component and mainly represented by imbricataloic acid, totarol, and dehydroabietic acid (1.3%, 1.2%, and 0.3%, respectively) (Table S1). This propolis sample had also fatty acids [hexadecanoic acid (2.8%), octadecanoic acid (1.9%), octadecenoic acid (0.5%), and tetracosanoic acid (0.8%)] and aromatic acids [benzoic acid (1.2%)].

The values of MIC found for sample 1 are higher than those reported for ethanolic extracts of propolis from Taiwan and Iran where MIC values ranged from <3.75 to 60 *µ*g/mL and 205 *μ*g/mL, respectively [70, 71] and lower than that from Canada (MIC = 2.74 mg/mL) for* S. aureus* strains [72]. The antimicrobial activity of propolis extracts has been attributed to a synergism effect of phenol compounds, including caffeic acid and caffeic acid phenetyl ester, and flavonoids [40]. However, in sample 1, diterpenes predominate, mainly isocupressic acid [58]. The results obtained may, therefore, be attributed to diterpenes along with phenols, by either synergism or antagonism. The antistaphylococcal activity of Mediterranean propolis, from Malta, was better than Bulgarian poplar propolis [73]. The authors, also, attributed this better activity to the possible presence of relative high amounts of diterpenes in Maltese propolis.

#### 3.3.2. Adaptation to Subinhibitory Concentrations of Propolis Extract on the Induction of Resistance to Propolis Extract or Antibiotics

In order to evaluate the impact of sequential exposure of* S. aureus* strains to propolis on developing resistance against this natural product, the effect of the adaptation of the* S. aureus* strains on the susceptibility to propolis extract was investigated.* S. aureus* ATCC 6538 was able to overcome just four passages at the MIC value (0.36 mg/mL) of propolis extract after the sequential passages at 0.12, 0.24, and 0.36 mg/mL, but failed to overcome the following lethal concentration of 0.49 mg/mL, after the first passage (Table 5). Adapted cells of MRSA15 and MRSA16 strains were able to overcome just four passages of the concentration 0.49 mg/mL after the same sequential passages. The MRSA 2 cells were able to overcome the four passages at 0.49 and 0.73 mg/mL. According to the EUCAST breakpoint tables (2016), the four* S. aureus* strains showed a susceptible profile to chloramphenicol, gentamicin, tetracycline, and erythromycin after the adaptation with different concentration of propolis (data not shown).

Beyond the antimicrobial activity of propolis extracts against* Staphylococcus* sp. and other microorganisms, they have also been reported to potentiate the action of antibiotics [74]. Propolis is widely used as ingredient in chewing gums, toothpastes and oral sprays, shampoos, soaps, and cosmetics. The repeated utilization of these products by people leads to a continuous exposure of microorganisms, including pathogens when an infectious disease is declared, to the subinhibitory concentrations of propolis. With this vast utilization of propolis under several forms, it is very important to clarify the impact of this repeated exposure of pathogens to propolis and their constituents on the development of resistance, to either propolis or their components and antibiotics. This sort of studies has been carried out by some authors [60, 75, 76] but with essential oils and/or their main components and controversial results have been reported. In the present work, it is clear that the sequential exposure to sublethal doses of propolis from Morocco did not result in the development of resistance to the propolis itself or to antibiotics (chloramphenicol and tetracycline for all strains; an erythromycin for* S. aureus* ATCC6538 and MRSA2). With such results, care must be taken with the frequent utilization of these kinds of natural products associated with some antibiotics, in the presence of particular MRSA strains.

#### 3.3.3. Influence of Propolis Extract on Adherence

**Figure 3:**
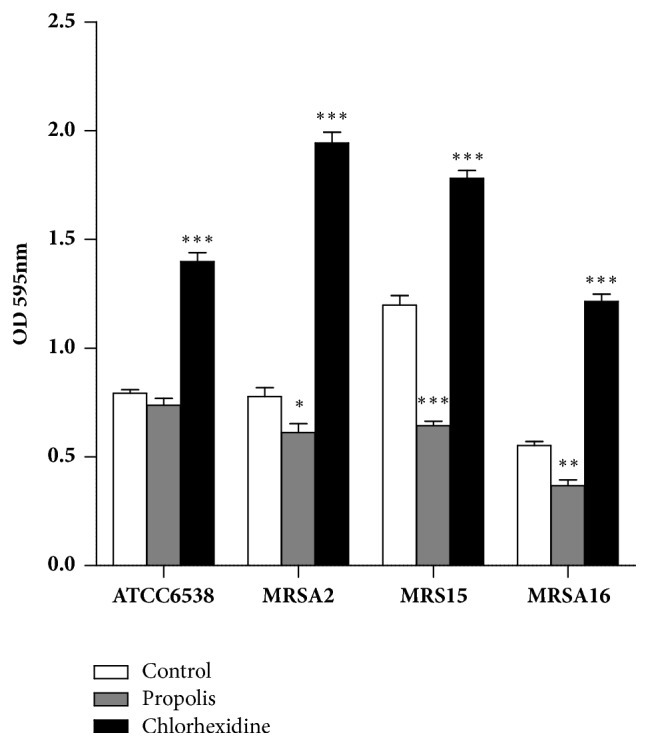
Impact of propolis extract (sample 1) at the MIC value (0.36 mg/mL) on the adherence ability of four* Staphylococcus aureus* strains. Data are the mean of three independent experiments (n = 12). Error bars represent the standard deviation. *∗ p* < 0.05; ∗∗* p* < 0.01; ∗∗∗* p* < 0.001, statistically significant when compared with the control.

The results of the impact of propolis on the adherence ability of* S. aureus* strains are illustrated in Figure 3. The adherence of the cells of ATCC 6538 strain was not affected by propolis exposure in comparison to the MRSA strains whose adherence was significantly impaired. In contrast, the exposure to chlorhexidine (0.2%,* w/v*) not only did not inhibit the adherence of all the* S. aureus* strains, but have boosted it significantly (*p* < 0.001) in comparison with the control. This finding is particularly interesting since a decrease in the susceptibility of clinical isolates of* S. aureus* has been reported [77, 78]. The adherence inhibition of MRSA strains by propolis was strain dependent, and the adherence of MRSA15 was particularly affected (*p* < 0.001) in comparison with the control. The adherence of MRSA16 was also impaired by the propolis extract (*p* < 0.01), but the adherence inhibition of the strain MRSA2 strain was lower (*p* < 0.05).

The ability to adhere of eight* S. aureus* strains was impaired in the presence of several concentrations of ethanolic extracts of propolis from Romania [12]. The prevention of biofilm formation promoted by propolis was also found for other microorganisms being also such property dose-dependent [12, 79, 80]. The geographic origin of propolis is another factor responsible for the variability of inhibition of biofilm formation, that is, dependent on the chemical composition. Those authors related the activities found with the chemical amounts of several types of phenols present in propolis samples. In contrast to the results reported by these authors, in the present work the presence of propolis extract did not impair the adherence of* S. aureus* ATCC6538 cells; nevertheless the diverse strains of MRSA cells were inhibited to form biofilm in the presence of propolis of the same origin. Diterpenes along with phenols, the main group of compounds of our extract of propolis (sample 1), may have a role in the activity found.

Bacterial biofilm formation is linked to the quorum-sensing system (QS), a cell-cell communication system that modulates gene expression and works in a cell density dependent mode [81]. In staphylococci, the QS mechanism comprises the accessory gene regulator (*agr*) locus that controls the production of a small auto-inducing signal peptide (AIP) [82]; how propolis impairs biofilm formation by disrupting the QS system in* S. aureus* needs further investigation.

#### 3.3.4. Screening of Propolis Extract for Anti-Quorum Sensing Properties

**Figure 4:**
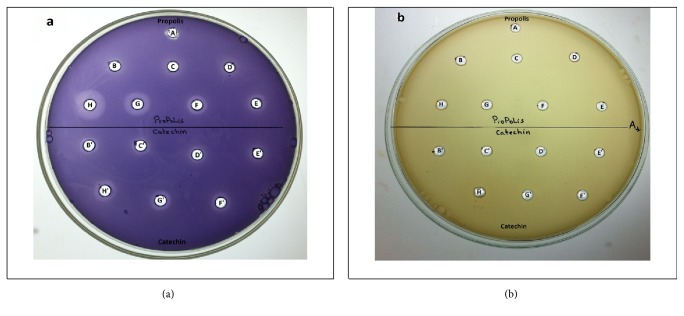
Anti-QS properties of propolis and catechin. (a) N-hexanoylhomoserine lactone (C6-HSL) at 0.12 *µ*g/mL was added to the culture medium. (b) No addition of C6-HSL to the culture medium. In each well was added (**A**): ethanol 70%; (**B**): propolis at 0.24 mg/mL; (**C**): propolis at 0.36 mg/mL; (**D**): propolis at 0.49 mg/mL; (**E**): propolis at 0.61mg/mL; (**F**): propolis at 0.73 mg/mL; (**G**): propolis at 0.98 mg/mL; (**H**): propolis at 1.22 mg/mL. (**B'**): (+)-catechin at 0.24 mg/mL; (**C'**): (+)-catechin at 0.36 mg/mL; (**D'**): (+)-catechin at 0.49 mg/mL; (**E'**): (+)-catechin at 0.61mg/mL; (**F'**): (+)-catechin at 0.73 mg/mL; (**G'**): (+)-catechin at 0.98 mg/mL; (**H'**): (+)-catechin at 1.22 mg/mL. This assay was conducted in three independent triplicates (N=9).

The anti-QS activity of propolis extract was determined by assessing the violacein production by* C. violaceum* and the results are shown in Figures 4(a) and 4(b). Loss of the purple pigment in* C. violaceum* indicates the inhibition of QS by the propolis extract. Control wells containing catechin and ethanol were included. The seven concentrations 0.24; 0.36; 0.49; 0.61; 0.73; 0.98; and 1.22 mg/mL showed halo zone on the purple background. Therefore, the highest inhibition zone (15.43±1.57mm) was observed for the concentration 1.22 mg/mL followed by 10.5±1.00 mm for the concentration 0.98 mg/mL. No inhibition was apparent with ethanol (Figure 4(a)).

Figure 4(b) showed no growth inhibition zone at all tested concentration of propolis; this demonstrates that propolis extract does not affect the growth of* C. violaceum* CV026.

QS inhibitory activity was already reported for distinct propolis samples from the United States, being such activities dependent on the geographic origin of propolis [83]. The authors considered that pinocembrin present in poplar propolis was a potential active compound on the QS inhibitory activity. However, in South African propolis, [84] reported that only caffeic acid had anti-quorum sensing property, being the remaining compounds present in propolis, including pinocembrin and their derivatives, unable to inhibit the violacein production, and therefore without anti-quorum sensing activity. Other studies showed that an isoprenyl caffeate-rich fraction of manuka propolis was particularly an active QS inhibitor [13]. These authors found that 1 mg dry weight/disc or at least 0.450 mg/mL of that fraction of manuka propolis completely inhibited violacein production, concluding the authors that the presence of isoprenyl caffeate was very important on the activity detected, because the activities found by other authors [85, 86] in distinct propolis samples were drastically inferior to those found by them. In the present work, isocupressic acid (8.1%), a diterpene, was the major compound of Moroccan propolis, immediately followed by pinocembrin (7.4%). Both compounds alone or along with other compounds present in the sample may have been responsible for the activity found.

#### 3.3.5. Influence of Propolis Extract on Virulence

**Figure 5:**
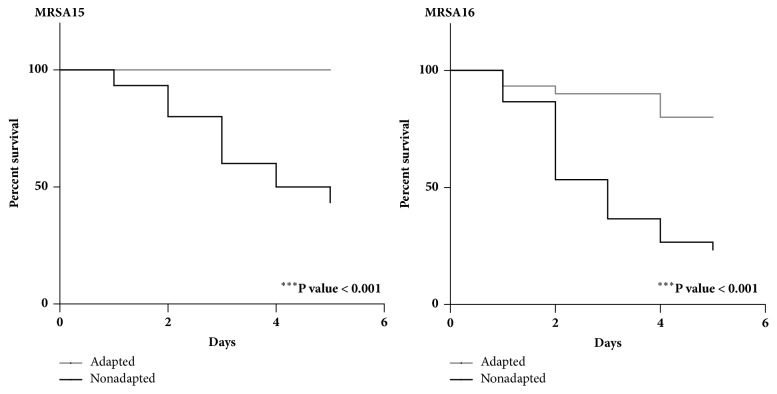
Kaplan–Meier survival curves for insects after injection with adapted cells (three passages at subinhibitory propolis concentration (0.24 mg/mL)) and nonadapted cells (three passages in BHI only, control). Test was done with sample 1. Data are the mean of three independent experiments (n = 30). Larvae injected with PBS showed a 100 % over the experiment.

The impact of adaptation on virulence of* S. aureus* strains was evaluated with the* G. mellonella* model. Adapted MRSA15 and MRSA16 strains submitted to subinhibitory propolis concentration were injected in* G. mellonella* larvae, and their survival was monitored (Figure 5). Differences in the ability of MRSA15 and MRSA16 to cause the death of the larvae were observed.

It was found that larvae injected with MRSA15 adapted cells presented 100% of survival in comparison with 43% of survival of insects injected with nonadapted cells (*p* < 0.001). MRSA15 was the less virulent strain in contrast to MRSA16 that caused a mortality of 20% of larvae injected with nonadapted cells by the end of the experiment; also significant differences (*p* < 0.001) in mortality of insects were observed between adapted and nonadapted cells. Larvae injected with PBS achieved a 100% survival until the end of the experiment.

The* G. mellonella* model has been used to evaluate the virulence potential of several human pathogens, including* S. aureus* and MRSA strains [60, 87]. This choice to use this model was also based on the fact that propolis has practically no toxic effect on the insect [88], although other studies had demonstrated the opposite, particularly for Chinese and Egyptian propolis extracts [89]. The results of the present work show that sequential exposure of MRSA strains to subinhibitory propolis concentration affected their virulence. Such data evidence that Moroccan propolis with diterpenes as main group of secondary metabolites has capacity for combating MRSA strains diminishing their virulence potential, inhibiting the formation of biofilm.

The screening of different Moroccan propolis samples regarding their antioxidant activities has revealed different antioxidant activities; these differences can be attributed to the compounds with antioxidant capacity in each propolis sample. This finding also illustrates the diversity of propolis and presents propolis as an important source of exogenous antioxidant that can be highly involved in the ROS related human diseases. On the other hand, we report in this work the efficiency of one of the most active propolis samples, as antibacterial agent against* Staphylococcus aureus* strains. Propolis did not induce the development of resistance to antibiotics, inhibit the biofilm formation, possess an anti-quorum sensing, and diminished the virulence of* S. aureus* for all tested strains.

It is clearly concluded that propolis from Morocco can be used in different field, as antioxidant source in order to use it on the benefit of human health and well-being. Also, being a natural product, it can be a brilliant candidate for biomedical application to combat nosocomial disease and the biofilm formation on medical device caused by* S. aureus.*

## Figures and Tables

**Table 1:** Phenols, flavones, and dihydroflavonols contents of hydro-alcoholic extracts of Moroccan propolis harvested at different places (adapted from [45]).

**Sample**	**Region **	**City**	**Surrounding plants**	**Phenols (mg Eq Ferulic Acid / g de propolis)**	**Flavones (mg Eq Quercetin / g de propolis)**	**Dihydroflavonols (mg Eq Eriodictyol / g de propolis)**
1	Fez-Boulemane	Sefrou	*Olea, Pinus, Quercus, Juniperus, Rosmarinus, Cistus, Lavandula and Pistacia.*	205.82±0.47^b^	53.50±0.36^c^	7.69±0.04^b^

2	Gharb-Chrarda-Beni-Hsen	Moulay.Bouselhame	*Vaccinium*	16.92±0.47^l^	2.90±0.36^jkl^	1.41±0.04^m^

3*∗*	Gharb-Ghrada-Beni-Hsen	Sidi Slimane	*Citrus,Zizifus, Silybum, and Ceratonia*	33.01±0.47^i^	10.57±0.36^g^	2.75±0.04^h^
		Khenichat				
	Meknes-Tafilalet	Khenifra				
	Tadla-Azilal	Beni mellal				

4	Meknes-Tafilalet	Khenifra	*Seratonia*	13.07±0.47^m^	2.63±0.36^kl^	1.48±0.04^m^

5	Meknes-Tafilalet	Khenifra	*Seratonia*	21.95±0.47^k^	4.29±0.36^ij^	2.08±0.04^k^

6	Doukkala-Abda	Oualidia	*Eucalyptus*	14.85±0.47^m^	3.21±0.36^jk^	1.80±0.04^l^

7	Souss-Massa-Draa	Zagora	*Thymus, Euphorbia and citrus*	7.80±0.47^no^	0.85±0.36^mno^	0.92±0.04^o^

8	Rabat-Sale-Zemmour-Zaer	Rabat	*Eucalyptus, Quercus and * Juniperus	57.62±0.47^f^	12.05±0.36^f^	2.51±0.04^i^

9	Gharb-Ghrada-Beni-Hsen	Moulay.Bouselham	Vaccinium	13.30±0.47^m^	1.72±0.36^lmn^	1.71±0.04^l^

10	Marketed in Morocco	Marketed in Morocco	Marketed in Morocco	144.07±0.47^c^	23.44±0.36^d^	5.03±0.04^e^

11	Taza-Al Hoceima-Taounate	Taza	*Olea, Pinus, Quercus, Juniperus, Rosmarinus, Cistus, Lavandula and Pistacia*	20.97±0.47^k^	2.01±0.36klm	1.80±0.04^l^

12	Unknown	Unknown	*Unknown*	9.53±0.47^n^	0.16±0.36^o^	1.13±0.04^n^

13	Gharb-Chrada-Beni-Hsen	Zeggouta	*Olea, Ceratonia, Chamaerops, Euphorbia, Juniperus, Quercus, Pinus, Pistacia, Thuya, and Eucalyptus*	39.20±0.47^h^	5.59±0.36^i^	2.14±0.04^jk^

14	Gharb-Chrada-Beni-Hsen	Sidi Slimane	*Eucalyptus*	63.33±0.47^e^	12.26±0.36^f^	4.17±0.04^f^

15	Rabat-Sale-Zemmour-Zaer	Temara	*Olea, Ceratonia, Chamaerops, Euphorbia, Juniperus, Quercus, Pinus, Pistacia, Thuya, and Eucalyptus*	57.86±0.47^f^	9.93±0.36^gh^	5.27±0.04^d^

16	Gharb-Chrada-Beni-Hsen	Kenitra	*Olea, Ceratonia, Chamaerops, Euphorbia, Juniperus, Quercus, Pinus, Pistacia, Thuya, and Eucalyptus*	53.61±0.47^g^	8.92±0.36^h^	4.26±0.04^f^

17	Fez Boulemane	Outat el Haj	*Olea, Pinus, Quercus, Juniperus, Rosmarinus, Cistus, Lavandula and Pistacia*	265.37±0.47^a^	129.60±0.36^a^	6.67±0.04^c^

18	Grand-Casablanca	Casablanca	*Thymus -euphorbia – Citrus *	24.04±0.47^j^	4.09±0.36^j^	1.74±0.04^l^

19	Rabat-Sale-Zemmour-Zaer	Tifelt	*Ricinus- chardon- Vaccinium -eucalyptus - Marrubium and Mentha*	14.30±0.47^m^	2.05±0.36^klm^	2.21±0.04^jk^

20	Fez -Boulmane	Sefrou	*Biplorum and Thymus*	72.33±0.47^d^	18.12±0.36^e^	3.26±0.04^g^

21	Guelmim-Es Semara	Tantan	*Arganier, Acacia, and Euphorbia*	7.83±0.47^no^	0.61±0.36^mn^	2.29±0.04^j^

22	Fez -Boulmane	Moulay Acoub	*Olea, Pinus, Quercus, Juniperus, Rosmarinus, Cistus, Lavandula and Pistacia*	56.75±0.47^f^	12.66±0.36^f^	4.14±0.04^f^

23	Marketed in Morocco	Marketed in Morocco	Marketed in Morocco	264.80±0.47^a^	80.97±0.36^b^	15.95±0.04^a^

24	Souss-Massa-Draa	Tiznit	*Citrus, Arganier, Acacia, and Euphorbia*	7.06±0.47^o^	1.17±0.36^mno^	1.74±0.04^l^

Values in the same column followed by the same letter are not significantly different (*p *<0.05) by Tukey's multiple range test. *∗*The beehives were displaced between the regions reported above, according to the information of the beekeeper.

**Table 2:** Antioxidant activity of hydro-alcoholic extracts of Moroccan propolis harvested at different places, measured through distinct methods, and expressed in IC_50_ (mg/mL), and Trolox equivalent (TE) for ORAC assay and mgequivalent ascorbic acid/g sample (mg Eq A.Asc/g) (mean ± standard error).

	**DPPH (mg/mL)**	**ABTS (mg/mL)**	**Molybdate (mg Eq A.Asc/g)**	**NO (mg/mL)**	**Chelating (mg/mL)**	**Superoxide (mg/mL)**	**H**_**2**_ **O**_**2**_ ** (mg/mL)**	**ORAC (**μ**mol TE/g)**
**1**	0.022±0.044^a^	0.014±0.123^a^	48.92±0.04^m^	0.310±0.195^abc^	2.429±0.291^fgh^	0.810 ±0.233^abc^	0.086±0.021^a^	1357.150±33.793^k^
**2**	0.605±0.044^fg^	0.310±0.123^ab^	29.57±0.0^g^	5.194±0.195^h^	1.157±0.291^abcde^	3.413 ±0.233^f^	ND	681.336±33.793^ab^
**3**	0.772±0.044^g^	0.215±0.123^ab^	32.57±0.04^h^	2.360±0.195^e^	1.151±0.291^abcd^	5.359 ±0.233^gh^	0.373±0.021^fg^	821.727±33.793^cdefg^
**4**	0.400±0.044^de^	0.182±0.123^ab^	24.69±0.04^d^	5.230±0.195^h^	1.583±0.291^cdef^	5.466 ±0.233^gh^	0.778±0.021^gh^	691.713±33.793^abc^
**5**	0.541±0.044^ef^	0.189±0.123^ab^	28.16±0.04^f^	3.758±0.195^g^	1.865±0.291^defg^	4.807 ±0.233^g^	0.608±0.021^gh^	720.261±33.793^abcd^
**6**	0.343±0.044^d^	0.388±0.123^ab^	16.73±0.04^b^	3.615±0.195^fg^	2.288±0.291^efgh^	ND	0.479±0.021^h^	698.592±33.793^abc^
**7**	1.935 ±0.044^i^	2.085±0.123^d^	9.40±0.04^a^	15.072±0.195^l^	1.325±0.291^abcdef^	ND	ND	652.953±33.793^a^
**8**	0.356±0.044^d^	0.321±0.123^ab^	20.48±0.04^c^	1.269±0.195^d^	2.879±0.291^gh^	1.139 ±0.233^bcd^	ND	801.962±33.793^bcdefg^
**9**	0.290±0.044^d^	0.144±0.123^ab^	33.57±0.04^h^	3.056±0.195^efg^	1.563±0.291^bcdef^	1.684 ±0.233^cde^	0.436±0.021^gh^	848.374±33.793^defgh^
**10**	0.024±0.044^a^	0.014±0.123^a^	39.39±0.04^h^	0.372±0.195^abc^	0.606±0.291^abc^	0.253 ±0.233^a^	0.168±0.021^bc^	1072.133±33.793^j^
**11**	0.386±0.044^de^	0.260±0.123^ab^	26.22±0.04^e^	3.429±0.195^fg^	1.204±0.291^abcde^	1.919±0.233^de^	0.928±0.021^gh^	954.108±33.793^hij^
**12**	1.160±0.044^h^	0.595±0.123^b^	21.34±0.04^c^	12.087±0.195^j^	1.074±0.291^abcd^	ND	ND	707.142±33.793^abc^
**13**	0.237±0.044^bcd^	0.058±0.123^a^	33.64±0.04^h^	2.425±0.195^e^	0.251±0.291^a^	0.723 ±0.233^ab^	0.463±0.021^h^	875.555±33.793^fgh^
**14**	0.101±0.044^abc^	0.057±0.123^a^	35.01±0.04^i^	0.795±0.195^bcd^	2.790±0.291^gh^	0.506 ±0.233^ab^	0.214±0.021^cd^	881.777±33.793^ghi^
**15**	0.107±0.044^abc^	0.017±0.123^a^	33.54±0.04^h^	1.344±0.195^d^	0.716±0.291^abc^	0.571 ±0.233^ab^	0.124±0.021^ab^	926.763±33.793^ghi^
**16**	0.101±0.044^abc^	0.044±0.123^a^	35.32±0.04^i^	0.822±0.195^bcd^	1.548±0.291^bcdef^	0.459 ±0.233^ab^	0.254±0.021^de^	918.095±33.793^ghi^
**17**	0.013±0.044^a^	0.009±0.123^a^	37.80±0.04^j^	0.025±0.195^a^	6.037±0.291^i^	0.323 ±0.233^ab^	ND	1723.289±33.793^l^
**18**	0.687±0.044^fg^	0.557±0.123^b^	20.73±0.04^c^	2.985±0.195^ef^	0.441±0.291^ab^	5.863 ±0.233^gh^	1.856±0.021^gh^	750.594±33.793^abcdef^
**19**	0.638±0.044^fg^	0.279±0.123^ab^	40.20±0.04^kl^	6.162±0.195^i^	0.963±0.291^abcd^	2.497 ±0.233^e^	0.473±0.021^h^	736.201±33.793^abcde^
**20**	0.260±0.044^cd^	0.259±0.123^ab^	27.58±0.04^f^	1.053±0.195^cd^	7.473±0.291^j^	4.880 ±0.233^g^	0.315±0.021^ef^	1005.958±33.793^ij^
**21**	3.110±0.044^j^	2.768±0.123^e^	40.89±0.04^l^	13.788±0.195^k^	3.054±0.291^h^	ND	ND	630.392±33.793^a^
**22**	0.077±0.044^ab^	0.049±0.123^a^	40.27±0.04^kl^	0.742±0.195^abcd^	10.495±0.291^k^	1.137 ±0.233^bcd^	0.290±0.021d^e^	851.107±33.793^efgh^
**23**	0.007±0.044^a^	0.002±0.123^a^	39.71±0.04^kl^	0.194±0.195^ab^	5.464±0.291^i^	0.213 ±0.233^a^	0.048±0.021^a^	1616.654±33.793^l^
**24**	1.793±0.044^i^	1.265±0.123^c^	23.43±0.04^d^	12.290±0.195^j^	0.881±0.291^abcd^	ND	ND	647.116±33.793^a^

Values in the same column followed by the same letter are not significantly different (*p*<0.05) by Tukey's multiple range test. ND: not detected at the concentrations tested.

**Table 3:** Spearman correlation coefficients among total phenols and flavonoids and antioxidant activities.

	**Total phenol**	**Flavone+Flavonol**	**Flavanone+Dihydroflavonol**
**Total phenol**	1	0.965^*∗∗*^	0.857^*∗∗*^
**Flavone+Flavonol**	0.965^*∗∗*^	1	0.849^*∗∗*^
**Flavanone+Dihydroflavonol**	0.857^*∗∗*^	0.849^*∗∗*^	1
**Molybdate**	0.458^*∗*^	0.440^*∗*^	0.705^*∗∗*^
**DPPH**	-0.855^*∗∗*^	-0.828^*∗∗*^	-0.773^*∗∗*^
**ABTS**	-0.691^*∗∗*^	-0.634^*∗∗*^	-0.645^*∗∗*^
**Chelating assay**	0.359^*∗*^	0.415^*∗*^	0.394^*∗*^
**Nitric oxide**	-0.951^*∗∗*^	-0.941^*∗∗*^	-0.851^*∗∗*^
**Hydrogen peroxide **	0.735^*∗∗*^	-0.807^*∗∗*^	-0.920^*∗∗*^
**ORAC**	0.868^*∗∗*^	0.791^*∗∗*^	0.778^*∗∗*^
**Superoxide scavenging**	-0.675^*∗∗*^	-0.567^*∗∗*^	-0.737^*∗∗*^

*∗* Correlation is significant at the *p* < 0.05 level. *∗∗* Correlation is significant at the *p *< 0.01 level.

**Table 4:** Susceptibility of *S. aureus* strains to propolis extracts using disc diffusion method. Minimum inhibitory (MIC) and minimum bactericidal concentrations (MBC) of propolis extracts.

**Strains**	**Origin**	** Source**	**0.73 mg/mL**	**0.98 mg/mL**	**Chloramphenicol**	**Ethanol**	**MIC (mg/mL)**	**MBC (mg/mL)**
**Sample 1**								
*S. aureus ATCC 6538*	Wound	American Type Culture Collection	22.33±0.57	27.00±1.52	26.00±2.51	--	0.36	0.98
*MRSA2*	Clinical	UAlg, CBMR. Portugal	19.67±1.15	25.67±0.57	19.00±0.57	--	0.36	1.22
*MRSA15*	Clinical	UAlg, CBMR, Portugal	26.67±1.51	31.67±1.73	24.33±0.01	--	0.36	1.22
*MRSA16*	Clinical	UAlg, CBMR, Portugal	25.67±0.05	32.33±1.51	24.67±2.29	--	0.36	1.22
**Sample 7**								
*S. aureus ATCC 6538*	Wound	American Type Culture Collection	12.33±2.30	14.33±0.57	25.00±3.08	--	ND	ND
*MRSA2*	Clinical	UAlg, CBMR. Portugal	14.00±4.35	16.33±4.93	22.67±2.04	--	ND	ND
*MRSA15*	Clinical	UAlg, CBMR, Portugal	15.00±1.01	16.00±1.01	25.67±1.52	--	ND	ND
*MRSA16*	Clinical	UAlg, CBMR, Portugal	15.67±0.57	17.67±2.88	21.33±1.52	--	ND	ND

--: no effect; ND: not determined.

**Table 5:** Impact of continued exposure of *Staphylococcus aureus* strains on increasing concentrations of propolis*∗*.

**Passages **	**Concentration of propolis sample (mg/mL)**				
	**0.12**	**0.24**	**0.36**	** 0.49**	**0.73**
***Staphylococcus aureus *ATCC 6538**					
**1**°	72.44±21.87^a^	42.81±1.41^a^	40.24±9.81^a^	14.22±0.43	NA
**2**°	36±10.72^a^	50.88±7.26^a^	61.33±10.00^a^	NG	
**3**°	87.17±61.12^a^	100.52±47.22^a^	57.33±15.19^a^		
**4**°	24.71±6.5^a^	103.68±16.5^a^	74.35±18.23^a^		
***Staphylococcus aureus *methicillin-resistant 2 (MRSA 2)**					
**1**°	114.09±35.26^a^	131.95±24.28^a^	5.33±0.91^a^	26.35±3.37^a^	35.92±18.65^a^
**2**°	139.7±45.72^a^	99.90±4.81^a^	48.49±15.95^b^	39.26±3.66^a^	8.98±3.02^a^
**3**°	75.38±13.02^a^	86.15±16.15^a^	55.75±24.95^b^	46.59±4.55^a^	31.46±26.68^a^
**4**°	105.17±21.68^a^	119.59±28.23^a^	33.20±3.82^ab^	83.55±20.67^b^	0.73±0.45^a^
***Staphylococcus aureus *methicillin-resistant 15 (MRSA 15)**					
**1**°	123.65±40.98^a^	125.40±15.72^b^	30.74±0.48^a^	32.74±27.93^a^	NA
**2**°	100.45±58.78^a^	124.64±9.27^b^	39.02±84.66^a^	27.13±7.29^a^	
**3**°	110.22±48.25^a^	123.99±36.06^b^	33.38±9.14^a^	18.38±15.47^a^	
**4**°	62.66±2.06^a^	65.66±11.69^a^	42.03±12.93^a^	0.59±0.40^a^	
***Staphylococcus aureus *methicillin-resistant 16 (MRSA 16)**					
**1**°	129.79±14.65^b^	43.57±6.84^a^	46.80±21.55^a^	43.22±8.18^c^	NA
**2**°	113.68±13.42^b^	96.39±13.47^b^	45.30±17.68^a^	22.09±10.66^bc^	
**3**°	57.71±7.81^a^	66.98±6.86a^b^	38.99±4.94^a^	21.60±9.03^b^	
**4**°	92.88±28.63^ab^	57.50±15.60^a^	83.80±29.52^a^	0.28±0.25^a^	

*∗*Fold change in growth at each passage is indicated as the ratio between the OD at T24 and the OD at T0 (ODT24/ODT0). **NG: **no growth; **NA**: not applied. Data are representative of three independent replicates. For each strain, data in the column with different letters are significantly different (*p* < 0.05).

## Data Availability

No data were used to support this study.

## Supplementary Materials

Supplementary Materials Table S1: chemical composition of hydro-alcoholic extract of Moroccan propolis.
