# Supplementary material for: Moroccan Propolis: A Natural Antioxidant, Antibacterial, and Antibiofilm against Staphylococcus aureus with No Induction of Resistance after Continuous Exposure
Source: Evid Based Complement Alternat Med. 2018 Nov 12;2018:9759240. doi: 10.1155/2018/9759240 (PMC6260532; doi:10.1155/2018/9759240)
Supplement: Supplementary Materials — Table S1: chemical composition of hydro-alcoholic extract of Moroccan propolis. [file 9759240.f1.doc]

**Moroccan propolis: a natural antioxidant, anti-bacterial and anti-biofilm against *Staphylococcus aureus* with no induction of resistance after continuous exposure**

**Soukaïna El-Guendouz^1,2^, Smail Aazza^1,2^, Badiaa Lyoussi^1^,Vassya Bankova^3^, Milena Popova^3^, Luís Neto^4^, Maria Leonor Faleiro^5^, Maria da Graça Miguel^2*^**

*^1^Laboratory of Physiology-Pharmacology-Environmental Health, Faculty of Sciences Dhar El Mehraz, BP 1796 Atlas, University Sidi Mohamed Ben Abdallah, Fez 30 000, Morocco*

*^2^Department of Chemistry and Pharmacy, Faculty of Science and Technology, University of Algarve, Campus de Gambelas, 8005-139 Faro, Portugal, MeditBio, 8005-139 Faro, Portugal.*

*^3^Institute of Organic Chemistry with Centre of Phytochemistry*, *Acad. G. Bonchev strl. bl. 9*, *1113 Sofia*, *Bulgaria*

*^4^Department of Biological Sciences and Bioengineering, Faculty of Science and Technology, University of Algarve, Campus de Gambelas, 8005-139 Faro, Portugal*

*^5^Department of Biological Sciences and Bioengineering, Faculty of Science and Technology, Center for Biomedical Research University of Algarve, Campus de Gambelas, 8005-139 Faro, Portugal, 8005-139 Faro, Portugal.*

|  | **Aromatic Acids** | **%** | **Diterpenes** | **%** | **Triterpenes** | **%** | **Sugars and Sugar Derivatives** | **%** | **Fatty Acids** | **%** |
| --- | --- | --- | --- | --- | --- | --- | --- | --- | --- | --- |
| **Sample 7** | Benzoic acid | 1.2 | Totarol | 1.2 | Lanosterol (3-α-OH) | 1.2 | Monosaccharides | 52.6 | Hexadecanoic acid | 2.8 |
|  | **Total** | **1.2** | Imbricataloic acid | 1.3 | α-amyrin | 0.4 | Disaccharides | 9.3 | Octadecanoic acid | 1.9 |
|  |  |  | Dehydroabietic acid | 0.3 | Lanosterol (3-β-OH) | 2.9 | Glycerol | 1.6 | Octadecenoic acid | 0.5 |
|  |  |  | **Total** | **2.8** | Lupeol | 3.5 | Inositol | 5.0 | Tetracosanoic acid | 0.8 |
|  |  |  |  |  | **Total** | **9.0** | **Total** | **68.5** | **Total** | **6.0** |
| **Sample 1** | (El-Guendouz et al., 2016a) | | | | | | | | | |
| Standard deviation does not succeed 6% for any of the constituents | | | | | | | | | | |

Table S1: Chemical composition of hydro-alcoholic extract of Moroccan propolis
